# The impact of time pressure on decision-making and visual search characteristics in basketball players

**DOI:** 10.3389/fpsyg.2025.1660732

**Published:** 2025-08-13

**Authors:** Zhi Guo, Qiulin Wang

**Affiliations:** College of Physical Education, Yangzhou University, Yangzhou, China

**Keywords:** basketball players, time pressure, visual search, eye movements, decision-making in sports

## Abstract

**Objective:**

This study aimed to investigate the effects of time pressure on decision-making and visual search behavior among college basketball players with different levels of expertise.

**Methods:**

Following the expert–novice paradigm, a total of 40 male participants were recruited, including 20 trained basketball athletes and 20 non-athlete college students. A 2 × 2 mixed factorial design was employed, with athletic expertise (athletes vs. non-athletes) as the between-subjects factor and time pressure (present vs. absent) as the within-subjects factor. Participants were presented with video clips from real basketball games. In the time pressure condition, they were required to make a decision—based on the actions of actual players in the video—within a limited time frame and complete a stress perception questionnaire. In the no time pressure condition, no time constraints were imposed. Decision-making performance (response time and accuracy) and eye-tracking metrics were recorded and analyzed.

**Results:**

(1) The expert group demonstrated significantly faster response times and higher decision-making accuracy compared to the novice group. (2) In terms of eye-tracking metrics, the expert group exhibited fewer fixations but significantly more saccades and longer fixation durations than the novice group. These findings suggest that experts engage in more focused and efficient visual processing with deeper cognitive engagement. (3) Analysis of fixation distribution and saccade trajectories revealed that experts concentrated more on key informational cues, with tighter and more centralized visual attention patterns, whereas novices showed more scattered and unfocused gaze behaviors.

**Conclusion:**

(1) Significant differences in visual search strategies were observed between athletes of different skill levels. Experts displayed more efficient search patterns, quicker responses, and superior decision-making performance. (2) Under time pressure, experts maintained high decision accuracy despite faster response times, and their eye movement patterns remained stable, indicating strong adaptability to stress. (3) In contrast, novices showed a marked decline in accuracy and visual search efficiency under time pressure, suggesting weaker cognitive control in high-stress situations.

## 1 Introduction

In competitive sports, time pressure is a typical situational factor that pervades various athletic disciplines. This is especially evident in basketball—a highly antagonistic, fast-paced, and decision-intensive team sport—where athletes are required to make rapid decisions within extremely limited time frames, involving information acquisition, situational assessment, and action execution (Bourgeais et al., 2025). Time pressure reflects an individual’s subjective psychological state under strict temporal constraints. Such high-pressure and stress-inducing competitive environments are inherent to basketball games and constantly influence athletes’ behavior (Giancamilli et al., 2022).

Decision-making is a goal-directed cognitive process in which individuals evaluate available information to make judgments and choices (Yuan et al., 2021). In the context of sports, this process is referred to as sport decision-making, which is defined as the comprehensive process by which athletes perceive, interpret, analyze, and respond to information in dynamic competitive environments (Yunchao et al., 2023). In basketball, sport decision-making is one of the core psychological and cognitive mechanisms that directly affects the execution efficiency of tactical strategies and the outcome of the game (Liu et al., 2024). Given the fast tempo of basketball, the frequent offensive-defensive transitions, and the rapidly changing on-court information, athletes must rapidly integrate and process complex spatial configurations, opponent movements, tactical cues, and their own physical states to make timely and accurate action choices (Hu, 2017). Particularly in decisive moments of a game, players often experience pronounced time pressure. Thus, the efficiency of decision-making in basketball is critical—not only in determining whether athletes make the right decision, but also whether they make it at the right time.

Time pressure refers to the sense of urgency experienced by individuals when time resources are limited or insufficient to accomplish a given task as planned (Kocher et al., 2019). It is a subjective cognitive response to the perceived scarcity of time and has been widely shown to exert significant influence on human cognitive behavior. Existing research indicates that time pressure can produce both negative and positive effects. On the one hand, it may lead to fatigue, anxiety, attentional lapses, and decreased efficiency (Jiang et al., 2024). On the other hand, it can, in the short term, stimulate potential, enhance attentional focus, and accelerate reaction speed, thereby improving task performance (Hsiao et al., 2017). Li et al. (2015) argued that time pressure exhibits a distinct “double-edged sword” characteristic, capable of both facilitating task completion and undermining task quality. When sufficient time is available, individuals can fully integrate information and formulate optimal strategies. However, under time scarcity—especially when the remaining time is inadequate to complete the task—emotional disturbances induced by time pressure may interfere with decision-making processes (Zheng et al., 2019). On the one hand, time pressure compresses the window for information processing, leading to restricted cognitive resource allocation and reduced decision accuracy (Quan, 2004; XiaoFei, 2024). Like other high-level cognitive functions, decision-making is prone to deterioration under psychological stress. Moreover, the need to process a large volume of information within a short time can result in information overload and lower processing efficiency, ultimately triggering cognitive biases (Du and Chen, 2023). On the other hand, some studies suggest that under certain conditions, time pressure may actually enhance decision quality. For example, research by Dawei (2009) demonstrated that time pressure can attenuate the framing effect, thus reducing decision-making biases and leading to more accurate outcomes. These findings confirm the dual nature of time pressure. However, whether this double-edged effect also manifests in the context of basketball remains unclear. The present study aims to explore how time pressure influences sport decision-making in basketball players.

In the context of sport decision-making, visual search plays a critical role. Visual search refers to the cognitive process by which individuals extract task-relevant information from complex environments through gaze behaviors such as fixations and saccades (Li et al., 2023; Wenfang et al., 2018). Given that more than 80% of external information humans receive is obtained through the visual system (Sireteanu and Rettenbach, 1995), an athlete’s ability to perceive and process spatial visual information largely determines their tactical judgment and motor responses. Previous studies have shown that basketball players’ decision-making behavior during games primarily depends on the real-time perception and processing of spatial information, most of which is acquired via the visual channel (JiaRan, 2024). Moreover, Jin et al. (2023) has reported that eye movement metrics during visual search can reflect the logical trajectory of athletes’ decision-making processes. Thus, understanding the visual search characteristics underlying sport decision-making is a prerequisite for studying such behavior. In recent years, the impact of time pressure on visual search performance has attracted growing attention. Xian-yu et al. (2021) found that time pressure exhibits an inverted U-shaped relationship with icon search performance, suggesting that moderate pressure may be most beneficial for enhancing task outcomes. Similarly, Lindong et al. (2016), through eye-tracking analyses, observed that increased time pressure leads to shorter fixation durations and changes in information processing patterns. These findings provide valuable insights into how time constraints may influence athletes’ visual search behaviors and decision-making mechanisms. However, it remains unclear how time pressure specifically affects visual search characteristics during decision-making in basketball players—a gap this study seeks to address.

In summary, building upon previous research, the present study introduces time pressure as a core variable to better simulate the real-game context of basketball and enhance the ecological validity of the investigation. Specifically, we aim to examine how time pressure influences the visual search characteristics (via eye-tracking indicators) and decision-making performance of collegiate basketball players with different skill levels. If time pressure is found to significantly affect decision-making, we can further explore how such effects manifest through changes in eye movement patterns. This study not only seeks to extend the application of time pressure research within the domain of sport-related cognitive behavior, but also aims to raise awareness among coaches and athletes regarding the importance of psychological skill training. Ultimately, the findings are expected to provide both theoretical insights and practical guidance for designing more scientifically grounded training protocols for university-level basketball teams—particularly those involving time-pressured scenarios.

## 2 Participants and methods

### 2.1 Participants

This study adopted the expert–novice paradigm widely used in sport psychology research on visual search (Peng et al., 2023). A total of 40 male basketball players were recruited from a university in Jiangsu Province, China. Participants were divided into two groups based on their skill level: an expert group (*n* = 20) and a novice group (*n* = 20). All participants were right-handed. The expert group comprised national first-level (or above) basketball athletes. The novice group consisted of basketball players with no specific ranking (see Supplementary Table S1). All participants had normal or corrected-to-normal vision, were in good physical and mental health, and demonstrated normal intellectual and psychological functioning. Participation was voluntary, and informed consent was obtained from all participants.

### 2.2 Experimental design

A 2 (Time Pressure: time pressure, no time pressure) × 2 (Expertise Level: expert group, novice group) mixed factorial design was employed. Expertise level (expert vs. novice) served as the between-subjects variable, and time pressure (with vs. without time constraint) as the within-subjects variable. The dependent variables included eye movement indicators (Annotation frequency, eye twitching frequency, fixation time, fixation trajectory chart, fixation heat map), decision accuracy, and reaction time. In the no time pressure condition, participants were allowed unlimited time to respond after the video ended and the decision screen appeared; the next trial began only after a response was made. In the time pressure condition, participants were required to make a decision within 1,000 milliseconds after the decision screen appeared. To ensure the effectiveness and validity of the time pressure manipulation, a Time Pressure Questionnaire was administered (see Supplementary Table S2). The questionnaire was originally developed by Svenson and later revised by to better reflect participants’ perceived pressure and internal state during task execution (Dawei, 2010). The revised version consists of eight items, and its Cronbach’s alpha coefficient was reported as 0.93, indicating high reliability and validity, meeting the requirements for psychological measurement.

### 2.3 Apparatus

The experiment was conducted using a Lenovo ThinkBook laptop with a 16-inch display and a screen resolution of 1920 × 1,080 pixels. Eye movement data were recorded using the Tobii Pro X3-120 eye tracker, developed by the renowned Swedish company Tobii Technology, with a sampling rate of 120 Hz. The Tobii Pro X3-120 is compatible with various screen types, including laptops and flat-panel monitors, and is suitable for a wide range of participant profiles, offering flexibility and precision in data collection across diverse experimental contexts.

### 2.4 Experimental materials

Decision-Making Videos: To ensure a high level of technical representation, all video materials were selected from games in the Chinese Basketball Association (CBA) league. To maintain video clarity and contemporary tactical relevance, matches were limited to recent seasons. Ultimately, games from the 2023 CBA season were chosen as the source. Following previous research on decision-making video stimuli and suggestions from experienced athletes, each video segment began at the initiation of an offensive play and was paused at the moment when the ball handler was stationary with possession, preparing to make a decision (Xiuying and Lizhong, 2017). A total of 83 video clips were selected for evaluation. These videos were distributed to basketball referees, master’s students specializing in basketball, and basketball coaches to assess and classify each video into one of three decision types: pass, drive, or shoot. During the video selection process, clips containing multiple decision points, complex scenarios, and critical or high-pressure moments were prioritized to increase the complexity of decision-making. Irrelevant videos were excluded, and the remaining videos were selected based on the experimental criteria. A total of 69 videos met the requirements, and 57 were ultimately chosen for the experiment. To maintain balance, each decision type was equally represented. The final selection included 19 shooting decision clips, 19 passing decision clips, and 19 driving decision clips.

### 2.5 Experimental procedure

**Figure 1:**
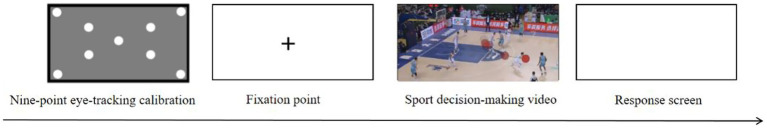
Sport decision-making procedure diagram.

The experimental procedure was programmed using the software provided with the Tobii Pro X3-120 eye tracker. As shown in Figure 1, the formal experiment consisted of 45 trials. In each trial, participants viewed a video clip depicting an offensive basketball scenario presented on the screen and were instructed to make a keypress decision simulating the most reasonable action the ball handler should take next. Participants chose among three options—pass, drive, or shoot—mapped to the J, K, and L keys on the keyboard, respectively. The video clips were presented in a randomized order. There was no time limit for responses during this phase; the next trial would appear once a decision was made. Throughout the experiment, the eye tracker automatically recorded participants’ reaction times, decision accuracy, and eye movement data related to visual search behavior.

### 2.6 Experimental procedure

(1) Each participant was individually brought into the laboratory. Prior to the experiment, participants were asked whether they experienced any physical discomfort. A folder named after each participant was created for storing and exporting their experimental data. The experimental equipment and ambient lighting in the room were then adjusted. The participant was provided with an overview of the experimental purpose and instructions, along with key points to note during the process.

(2) Participants were seated in an upright posture with their heads stabilized to minimize movement. They were instructed to maintain a viewing distance of 60 cm from the monitor and to focus their gaze on the screen. After head stabilization, the experimental instructions were displayed on the screen, followed by a nine-point eye-tracking calibration. Calibration was deemed successful if the average deviation was ≤ 0.5°. Upon successful calibration, participants rested with eyes closed for 30 s, after which they proceeded to a practice session consisting of 5 trials to familiarize themselves with the task procedure and keypress operations. A second calibration was performed following the practice session before the formal experiment commenced.

### 2.7 Data analysis

The Tobii Pro X3-120 eye tracker was used to collect data on decision-making accuracy, reaction time, and various eye movement indicators for each trial. All data were exported and initially processed using SPSS 26.0, including the removal of outliers and basic data cleaning. A two-way analysis of variance (ANOVA) was conducted to examine the main effects of expertise level and time pressure, as well as their interaction effects on the dependent variables.

## 3 Results analysis

### 3.1 Scores on the time pressure scale

Descriptive statistics of the participants’ scores on the time pressure scale were conducted (see Supplementary Table S3). Higher scores indicated a stronger perceived sense of time pressure. Both groups exhibited significantly higher time pressure scores under the time pressure condition compared to the no time pressure condition. These results confirm the effective manipulation of perceived time pressure during the experimental task.

**Figure 2:**
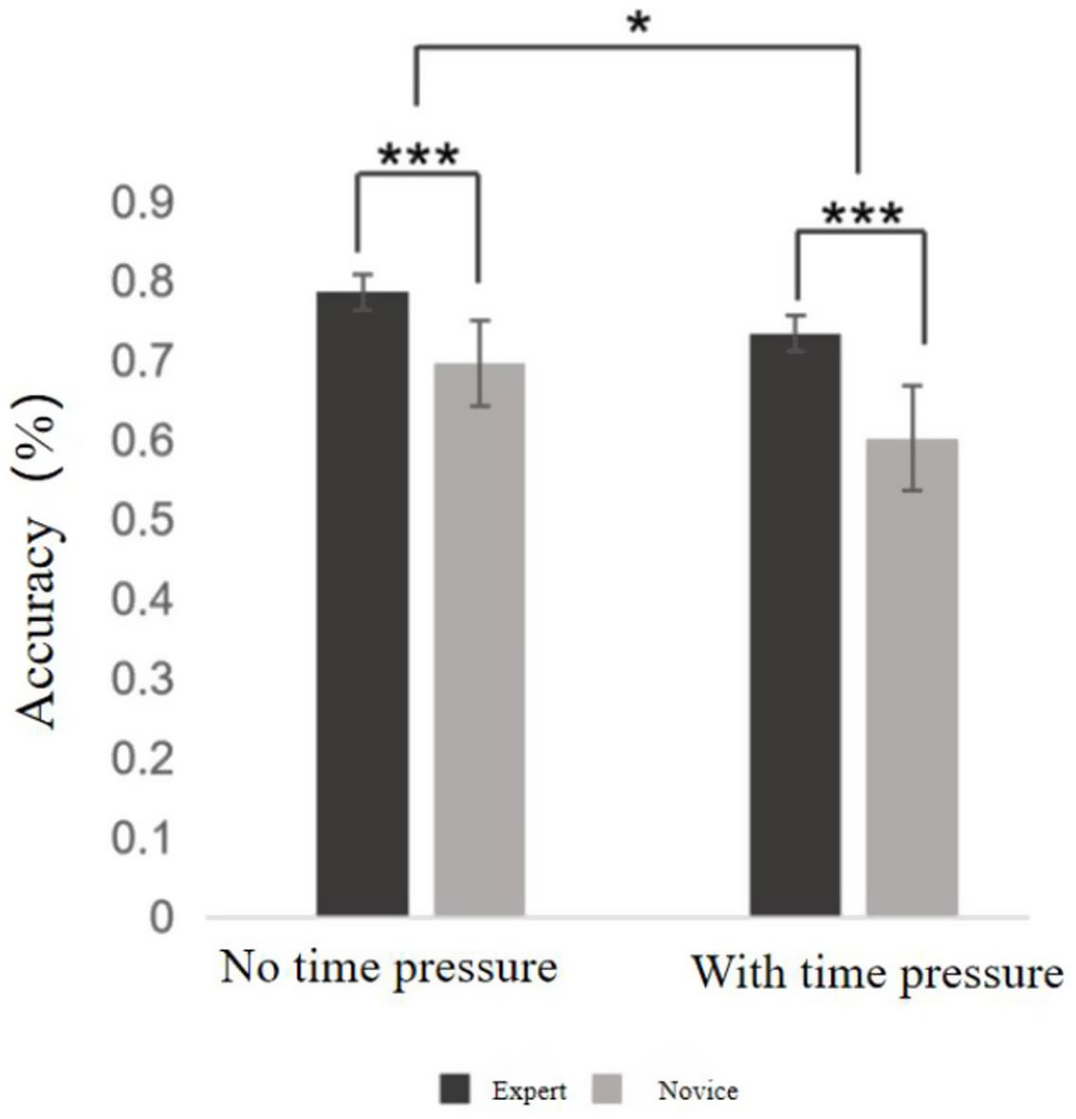
Decision-making accuracy results. ***Indicates *p* < 0.001, *indicates *p* < 0.05.

A two-way ANOVA was conducted on the time pressure scale scores of the two groups (see Supplementary Table S4). Results indicated that both group (expert vs. novice) and time pressure condition (presence vs. absence) had significant main effects on time pressure scores, while their interaction effect was not significant. This suggests that experts and novices differ in their perceived time pressure regardless of time pressure manipulation, and these effects arise independently rather than synergistically. Therefore, the time pressure manipulation implemented in this study holds meaningful relevance for investigating athletes’ responses (see Figure 2).

### 3.2 Decision-making accuracy

Descriptive statistics were first conducted to compare decision-making accuracy between expert and novice basketball players under both time pressure and no time pressure conditions (see Supplementary Table S5). A two-way ANOVA was then performed, revealing several significant effects (see Picture 99). A significant main effect of time pressure was observed, *F* = 77.622, *p* < 0.001, η^2^ₚ = 0.671, indicating that decision-making accuracy was significantly lower under time pressure than in the no-pressure condition across both groups. A significant main effect of skill level was also found, *F* = 85.937, *p* < 0.001, η^2^ₚ = 0.693, showing that expert players consistently outperformed novice players in decision accuracy, regardless of time pressure. Moreover, a significant interaction effect between time pressure and skill level emerged, *F* = 6.875, *p* = 0.013, η^2^ₚ = 0.153. This suggests that the impact of time pressure on decision-making accuracy differed depending on the athlete’s level of expertise. In particular, experts demonstrated greater resistance to performance decline under time constraints compared to novices.

Further simple effects analysis revealed that, for both groups, decision-making accuracy under time pressure was significantly lower than that under no time pressure (*p* < 0.001). This indicates that time pressure had a substantial negative impact on decision-making accuracy across all participants. However, the decline in accuracy was more pronounced among novice players, suggesting that less experienced athletes were more vulnerable to the detrimental effects of high-pressure situations.

### 3.3 Decision-making reaction time

**Figure 3:**
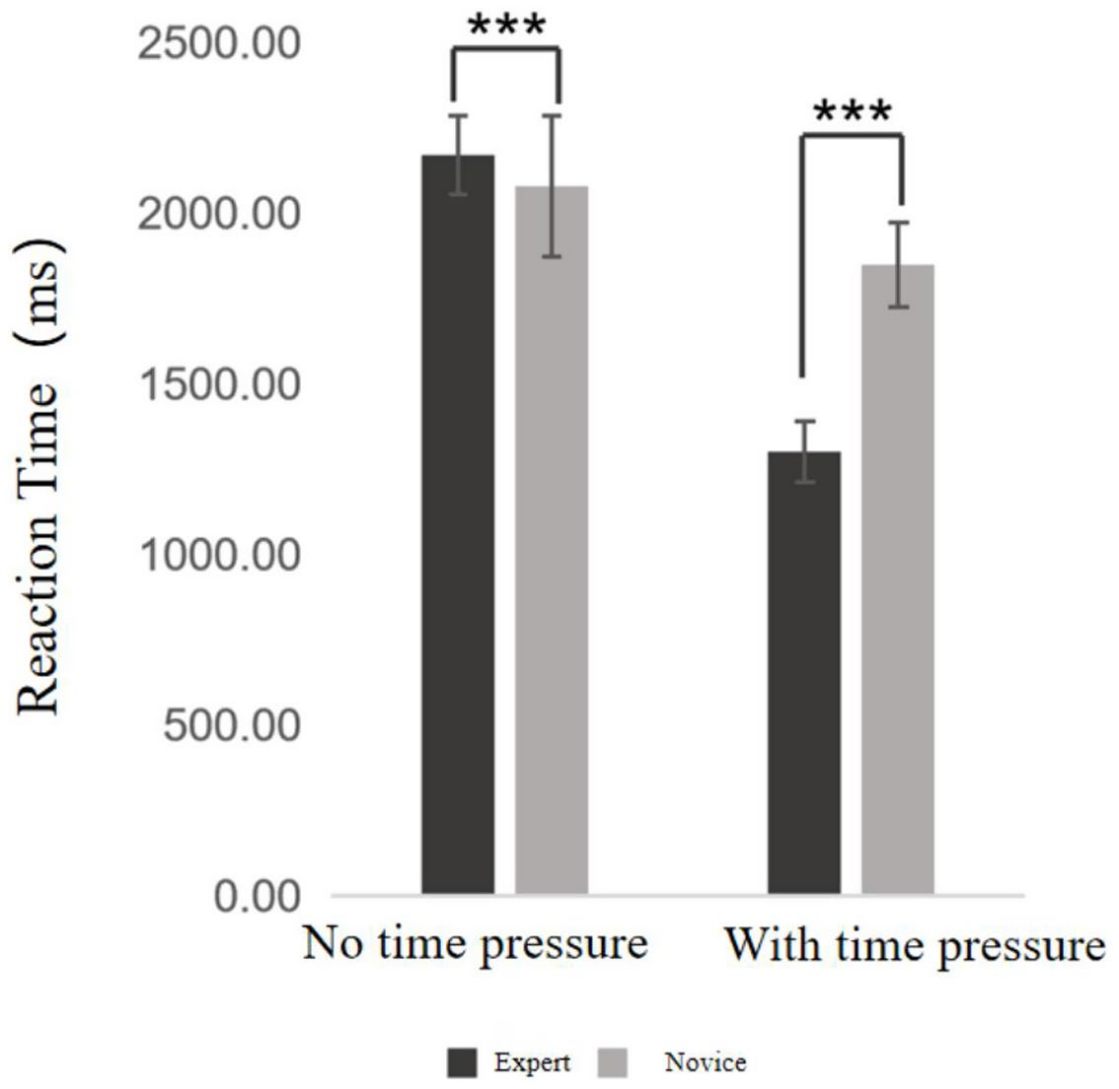
Decision-making reaction time results. ***Indicates *p* < 0.001.

Decision-making reaction time refers to the average amount of time taken by basketball players at different skill levels to complete each decision-making trial during the experiment. Descriptive statistics for expert and novice players under both time pressure and no time pressure conditions are presented in Supplementary Table S6. A two-way ANOVA was subsequently conducted, and the results (Figure 3) revealed the following: The main effect of time pressure was significant, *F* = 1298.483, *p* < 0.001, η^2^ₚ = 0.981, indicating a significant difference in reaction times between the two conditions. Specifically, decision-making was significantly faster under time pressure than in the absence of time pressure. The main effect of skill level was also significant, *F* = 186.779, *p* < 0.001, η^2^ₚ = 0.831, indicating that reaction times differed significantly between the expert and novice groups across both conditions. Expert players exhibited significantly faster reaction times than novice players. The interaction effect between time pressure and skill level was not significant (*p* > 0.05), suggesting that the two variables independently influenced reaction time, with no significant interaction effect.

### 3.4 Eye movement metrics analysis

Eye movement data for visual search were recorded using the Tobii Pro X3-120 eye tracker and subsequently imported into SPSS for statistical analysis. The selected eye-tracking metrics included fixation count, fixation duration, saccade count, as well as qualitative visualizations such as fixation scanpaths and heatmaps. These indicators collectively provided comprehensive insights into participants’ visual search behaviors during the task.

#### 3.4.1 Fixation count

**Figure 4:**
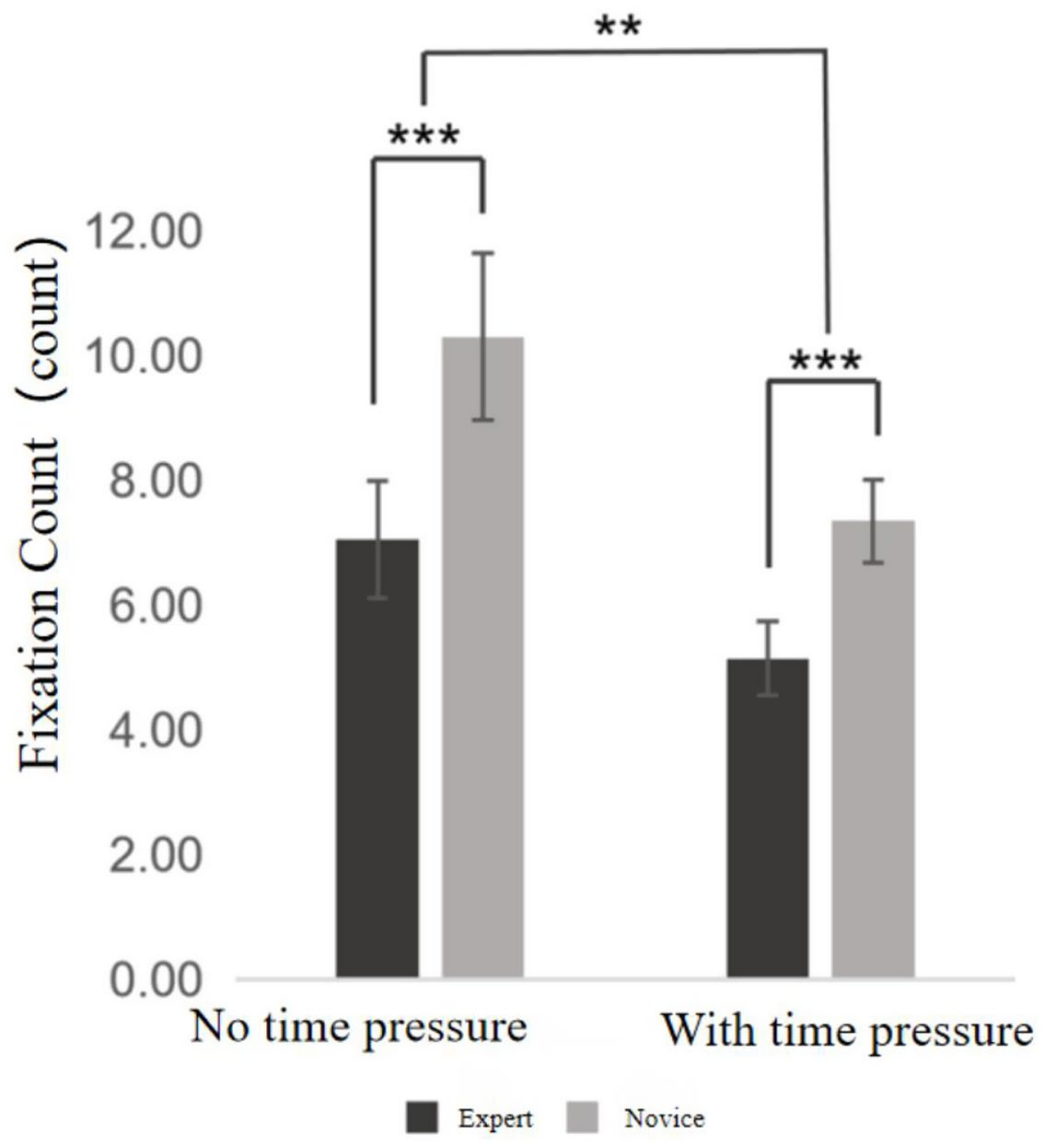
Fixation count chart. ***Indicates *p* < 0.001, **indicates *p* < 0.01.

Fixation count refers to the number of times the eyes fixate on a specific target during observation. The descriptive statistics of fixation count are shown in Supplementary Table S7. A two-way ANOVA was then conducted, and the results revealed the following (see Figure 4): The main effect of time pressure was significant, *F* = 163.261, *p* < 0.01, η^2^ₚ = 0.811, indicating that fixation count under time pressure differed significantly from that under no time pressure, with fewer fixations occurring under time pressure. The main effect of skill level was also significant, *F* = 145.170, *p* < 0.001, η^2^ₚ = 0.793, suggesting a significant difference in fixation count between expert and novice players under both time conditions, with experts exhibiting fewer fixations than novices. Furthermore, a significant interaction effect between time pressure and skill level was found, *F* = 7.562, *p* = 0.009 < 0.05, η^2^ₚ = 0.020, indicating that time pressure and skill level jointly influenced the fixation count.

Further simple effects analysis revealed that, for the expert group, fixation count under time pressure was significantly lower than under no time pressure (*p* < 0.001). Similarly, the novice group also exhibited significantly fewer fixations under time pressure compared to the no time pressure condition (*p* < 0.001). In the absence of time pressure, the expert group showed significantly fewer fixations than the novice group (*p* < 0.001); under time pressure, the expert group still demonstrated significantly fewer fixations than the novice group (*p* < 0.001). These findings suggest that time pressure had a greater impact on the novice group than on the expert group.

#### 3.4.2 Fixation duration

**Figure 5:**
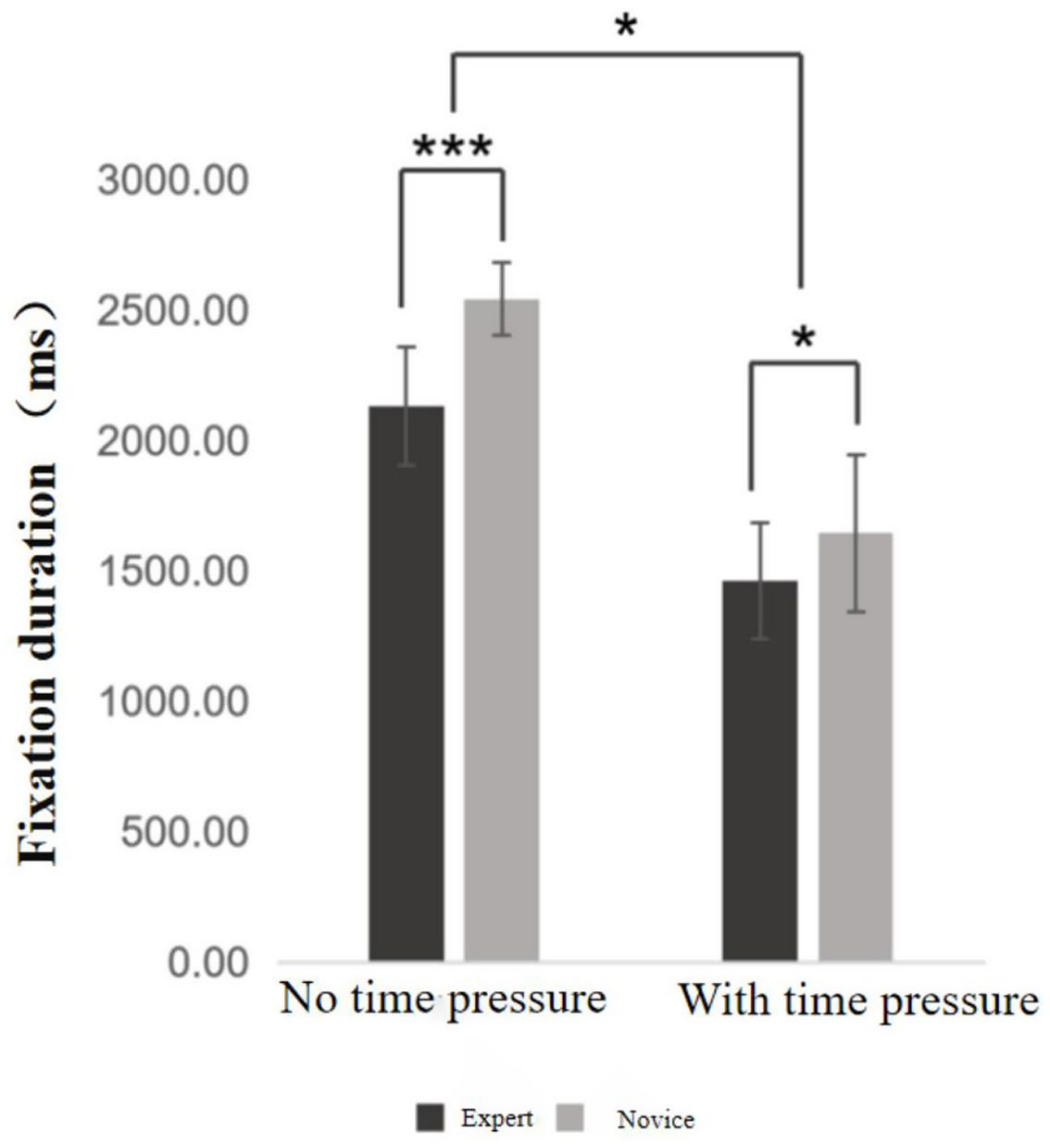
Fixation duration chart. ***Indicates *p* < 0.001, *indicates *p* < 0.05.

Fixation duration refers to the length of time participants maintain visual gaze on a specific target during the visual search task. This variable reflects the efficiency and speed of information processing when responding to visual stimuli. Descriptive results are presented in Supplementary Table S8. A 2 (time pressure: with vs. without) × 2 (skill level: expert vs. novice) mixed-design ANOVA was conducted to examine differences in fixation duration (see Figure 5). The results revealed a significant main effect of time pressure, *F* = 205.17, *p* < 0.01, η^2^ₚ = 0.844. Participants exhibited significantly shorter fixation durations under time pressure compared to the no-pressure condition. There was also a significant main effect of skill level, *F* = 31.53, *p* < 0.001, η^2^ₚ = 0.453. Across both conditions, expert basketball players demonstrated significantly shorter fixation durations than novice players, indicating a higher level of visual processing efficiency. Importantly, the interaction effect between time pressure and skill level was statistically significant, *F* = 4.86, *p* = 0.034, η^2^ₚ = 0.113. This suggests that the influence of time pressure on fixation duration was moderated by the participant’s level of expertise—expert players were less negatively affected by time constraints compared to novices.

Simple effects analysis revealed that within the expert group, fixation duration under time pressure was significantly shorter than in the no-pressure condition (*p* < 0.001). Similarly, novice players also showed significantly shorter fixation durations under time pressure compared to the no-pressure condition (*p* < 0.001). Between-group comparisons indicated that under the no-pressure condition, expert players exhibited significantly shorter fixation durations than novices (*p* < 0.001). This group difference remained significant under time pressure, although to a lesser extent (*p* = 0.035).

#### 3.4.3 Saccade count

**Figure 6:**
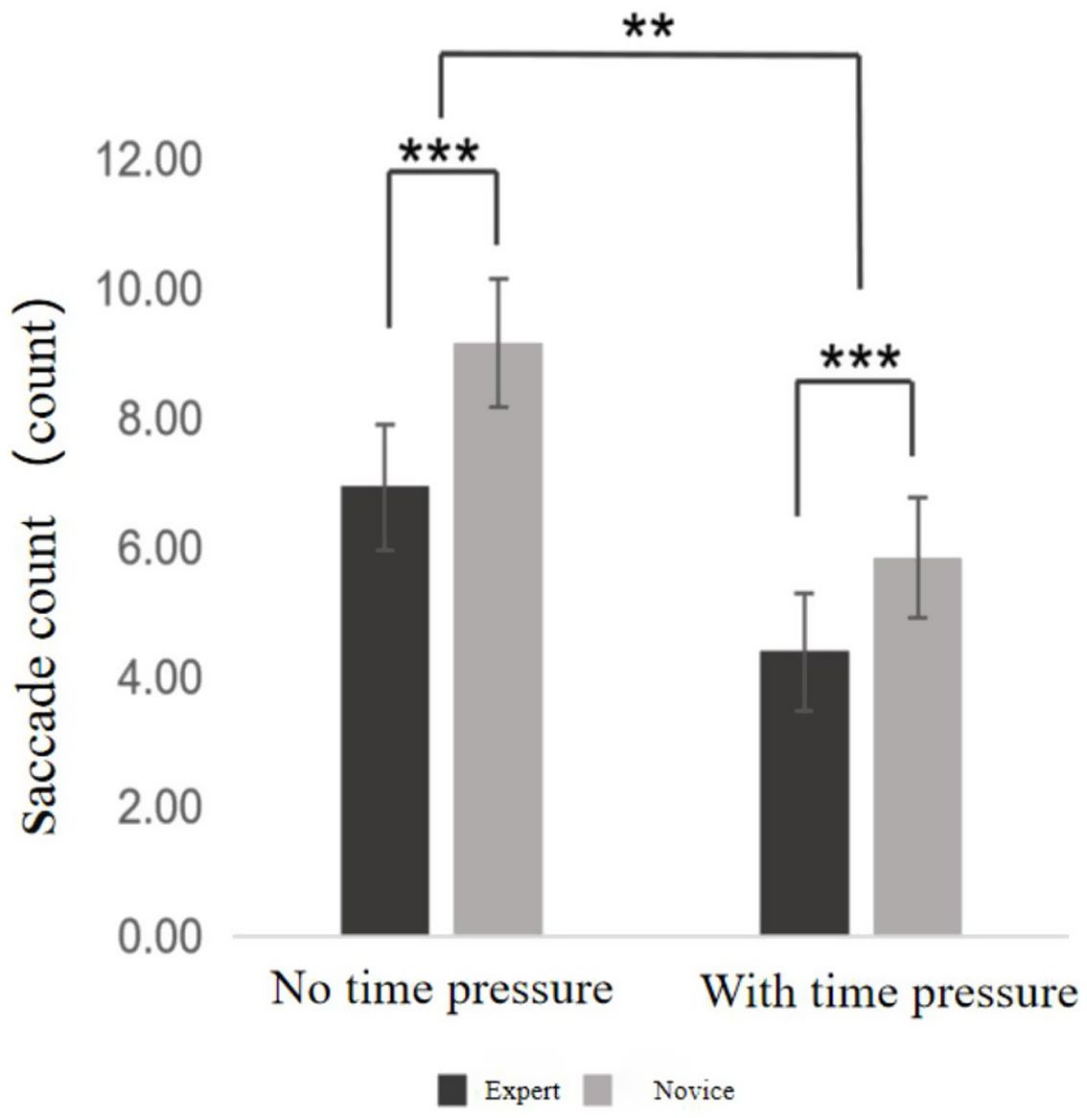
Saccade counts chart. ***Indicates *p* < 0.001, **indicates *p* < 0.01.

Saccades refer to rapid eye movements that shift central foveal vision from one point to another, typically resulting in fixation. In the context of this study, saccade count represents the number of foveal shifts made by participants during the visual search task. Descriptive statistics for saccade count are presented in Supplementary Table S9. A 2 (time pressure: with vs. without) × 2 (skill level: expert vs. novice) mixed-design ANOVA was conducted (see Figure 6). Results showed a significant main effect of time pressure, *F* = 614.87, *p* < 0.001, η^2^ₚ = 0.942, indicating that saccade counts under time pressure were significantly lower than in the no-pressure condition. A significant main effect of skill level was also found, *F* = 44.98, *p* < 0.001, η^2^ₚ = 0.542, demonstrating that, regardless of condition, expert players exhibited significantly fewer saccades than novices. Moreover, the interaction effect between time pressure and skill level was significant, *F* = 10.11, *p* = 0.003, η^2^ₚ = 0.210, suggesting that the impact of time pressure on saccade count varied according to participants’ level of expertise.

In the expert group, saccade counts under time pressure were significantly lower than those without time pressure (*p* < 0.001). Similarly, novices exhibited significantly fewer saccades under time pressure compared to no time pressure (*p* < 0.001). Moreover, experts demonstrated significantly fewer saccades than novices both in the absence (*p* < 0.001) and presence of time pressure (*p* < 0.001).

#### 3.4.4 Gaze trajectory map

**Figure 7:**
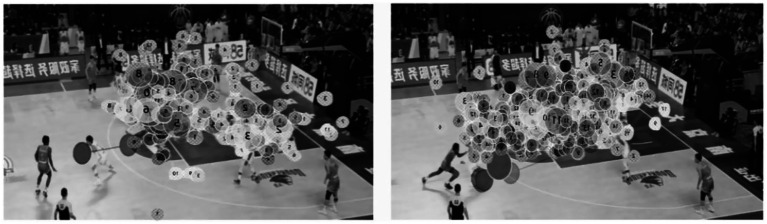
Gaze trajectory plots of expert (left) and novice (right) basketball players under no time pressure.

**Figure 8:**
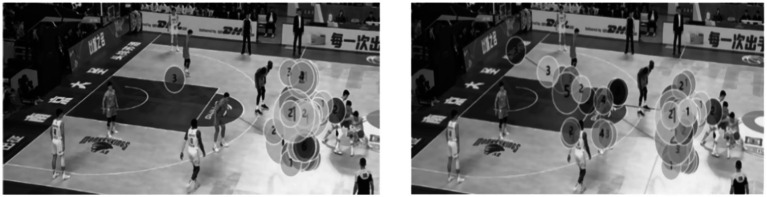
Gaze trajectory plots of expert (left) and novice (right) basketball players under time pressure.

The visualized gaze trajectory plots represent a single experimental trial comparing expert and novice basketball players under both time pressure and no time pressure conditions (see Figures 7, 8). Specifically, under the no time pressure condition, the expert group displayed highly focused fixation points, primarily concentrated on the off-ball defensive player area, reflecting strong tactical awareness and an overall grasp of game dynamics. In contrast, the novice group showed a more scattered distribution of fixation points, covering a broader area but lacking focus, indicating weaker strategic processing and global perception in visual information extraction. Under time pressure, experts were still able to maintain concentrated gaze patterns, suggesting stable and efficient information processing capabilities even in high-stress conditions. Conversely, the novice group exhibited increasingly dispersed gaze trajectories under time pressure, revealing poorer attentional control and less coherent, strategically guided visual behavior.

#### 3.4.5 Gaze heatmap

**Figure 9:**
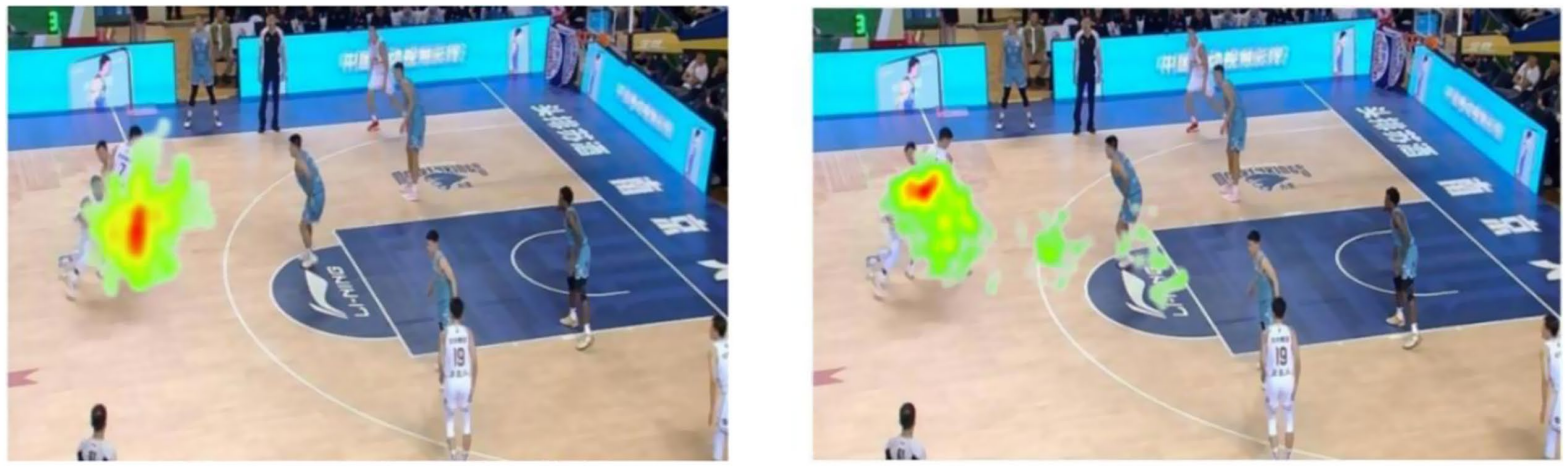
Gaze heatmaps of expert (left) and novice (right) basketball players under no time pressure.

**Figure 10:**
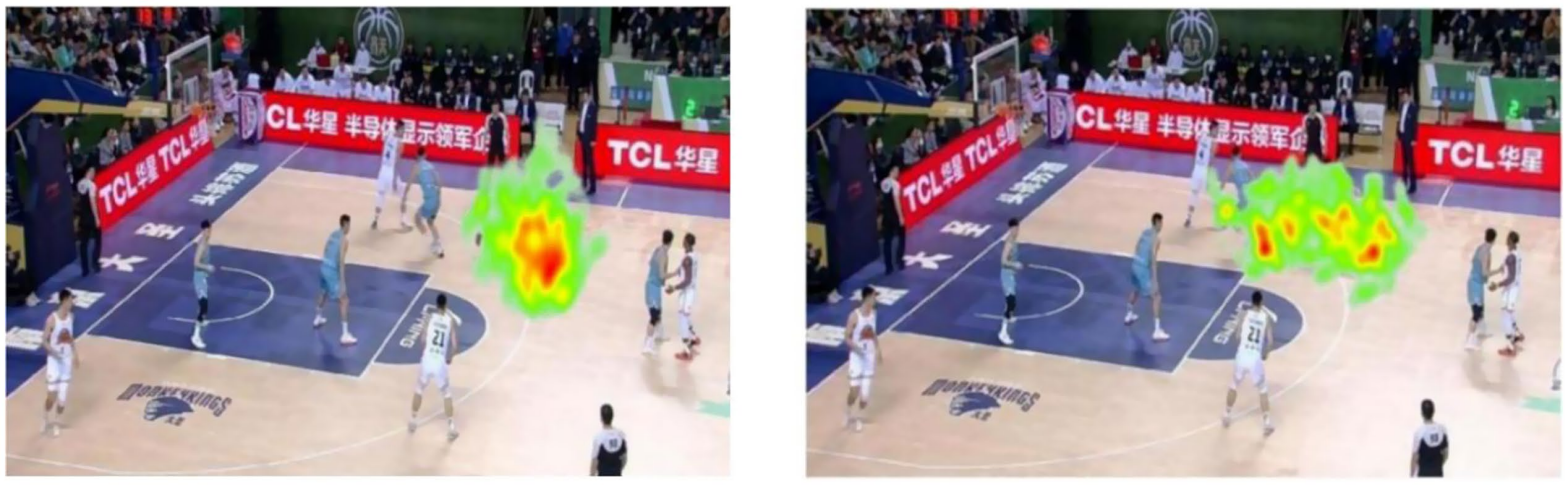
Gaze heatmaps of expert (left) and novice (right) basketball players under time pressure.

The visualized heatmaps illustrate the distribution of gaze fixations among expert and novice basketball players while viewing a dynamic game scenario video under time pressure and no time pressure conditions (see Figures 9, 10). The color gradient in the heatmap ranges from green (low fixation density) to red (high fixation density), indicating the concentration of visual attention. No Time Pressure Condition Experts: Fixations were highly concentrated in key decision-making areas, forming prominent red focal zones in the heatmap. This indicates that expert players allocated their visual attention efficiently and extracted task-relevant information with high precision. Novices: Fixation patterns were more scattered, with dominant green and yellow regions and limited red zones, suggesting unstable visual search strategies and broadly distributed but less efficient attentional allocation. Time Pressure Condition Experts: Although the red intensity slightly decreased compared to the no-pressure condition, focus remained within core task-relevant areas. This implies that expert athletes maintained effective attentional control and decision-making ability under stress. Novices: Gaze patterns became even more dispersed, with several green and yellow regions and a lack of central red zones. This suggests ineffective visual search strategies under time pressure, likely due to cognitive overload and impaired attentional focus. In summary, expert players exhibited consistent, focused, and efficient gaze behavior across both conditions, whereas novices showed dispersed and inefficient visual search, with greater impairment under time pressure. These findings highlight a skill-level difference in visual attention strategies, indicating that experts possess stronger resistance to pressure and superior attentional control during decision-making tasks.

## 4 Discussion

### 4.1 Differences in motor decision-making and visual search characteristics among college basketball players of varying skill levels

#### 4.1.1 Differences in motor decision-making

Under both time-constrained and non-time-constrained conditions, expert basketball players demonstrated superior decision accuracy and shorter reaction times compared to novices. This indicates that high-level athletes possess enhanced capabilities in information filtering and response control, enabling them to quickly extract critical information and make accurate judgments within limited timeframes. These findings support the experience-dependent nature of motor decision-making, aligning with the well-established “expert–novice paradigm” in the literature (Xiaoting et al., 2022). The expert group’s superior decision-making performance can be attributed to their long-term engagement in systematic training and extensive competitive experience. Over time, expert players develop stable knowledge structures and strategic schemas related to their sport, including technical-tactical knowledge, perceptual-cognitive skills, and psychological regulation strategies. These schemas allow them to efficiently activate stored information, extract and integrate relevant cues, and make rapid and effective decisions in dynamic game environments. In contrast, novice players often lack comprehensive training and game experience, resulting in fragmented and underdeveloped sport-specific knowledge. When facing complex, fast-paced scenarios, they struggle to identify key cues and are more likely to be distracted by irrelevant information. This leads to inefficient visual processing and redundant attention allocation, as evidenced by longer decision times and slower reactions, ultimately impairing both the accuracy and efficiency of their decisions. Prior studies also confirm that expert athletes accumulate substantial declarative and procedural knowledge in their domain, which facilitates more efficient cognitive processing and strategic execution during perception, judgment, and decision-making tasks (Jin et al., 2023; Nakamoto and Mori, 2008; Quan, 2004).

#### 4.1.2 Differences in visual search characteristics

The expert group outperformed the novice group across all eye-tracking metrics: they exhibited fewer fixations, shorter fixation durations, and fewer saccades. These findings align with previous research suggesting that experts rely more on intuitive judgments and possess a more structured perception of visual scenes, highlighting the critical role of experience in visual information processing. Moreover, their fixation heatmaps and scanpath diagrams were more focused and systematic. Heatmaps revealed the key areas of interest for expert players, while scanpath diagrams provided insight into their visual search strategies—explaining why their search behavior is more efficient (Alemanno et al., 2025). The superior visual search performance of expert athletes can be attributed to their long-term, systematic training and extensive competitive experience, which have led to the development of well-organized domain-specific cognitive structures in the brain (Yirui et al., 2024). These structures enable experts to rapidly recognize, encode, and extract critical contextual cues, resulting in faster and more accurate visual responses. Over time, they have developed a relatively mature and efficient visual search pattern that supports quick and precise decision-making under dynamic conditions. In contrast, novice players, lacking sufficient sport-specific knowledge and experience, tend to compensate for cognitive limitations by extending their search duration and increasing the number of fixations. This strategy allows them to gather more information in an attempt to maintain basic decision accuracy, but at the cost of efficiency. Their visual search tends to be less targeted and more scattered, reflecting a less refined perceptual-cognitive system.

### 4.2 The impact of time pressure on decision-making and visual search characteristics in basketball players

#### 4.2.1 The impact of time pressure on decision-making

The results of this study indicate that time pressure significantly affects both decision-making accuracy and reaction time in college basketball players. Specifically, time pressure led to a marked decline in decision accuracy across both expert and novice groups, suggesting that limited cognitive resources under high-pressure conditions increase the likelihood of judgment errors. This finding is consistent with Quan’s (2004) assertion that time pressure induces cognitive overload during information processing (XuYang, 2024). Conversely, decision reaction times were significantly reduced under time pressure, reflecting a “pressure-induced response acceleration” effect—where athletes are compelled to make rapid choices in pressing game situations, even at the expense of accuracy. This phenomenon exemplifies the classic speed–accuracy trade-off frequently observed in decision-making studies under pressure (Sha, 2022). Expert athletes, owing to their higher performance level and extensive competitive experience, have developed enhanced psychological regulation and a degree of adaptation to time-pressured environments. Combined with their refined sport-specific cognitive skills, they are able to maintain relatively high decision accuracy while simultaneously improving response speed (Nian et al., 2023). According to the challenge–hindrance stressor framework, time pressure is categorized as a challenge stressor, which is closely linked to an individual’s sense of task control and developmental potential. When managed appropriately, such stressors can enhance task motivation and facilitate performance through the mobilization of latent capabilities (Pearsall et al., 2009). Experts, supported by their superior self-regulation and well-developed cognitive schemas, generally possess a stronger sense of task mastery and performance potential. When facing time pressure, their motivation is further stimulated, allowing them to mobilize internal resources and optimize task execution. In contrast, novice players—lacking structured training and competition experience—have limited cognitive and emotional regulation capabilities and have yet to develop stable knowledge structures. Consequently, under high-pressure scenarios, they often experience a reduced sense of control and insufficient coping resources, leading to cognitive overload, emotional disturbance, and psychological stress. These conditions hinder their ability to attend to critical cues, ultimately compromising decision quality and task performance. This finding supports the dual-role perspective on time pressure proposed by Li et al. (2015), who argued that while time pressure can facilitate task completion, it may also impair task quality; the present study provides empirical support for this notion.

#### 4.2.2 The impact of time pressure on visual search characteristics

Eye-tracking analysis revealed that time pressure had a significant impact on all key visual search indicators, including the number of fixations, fixation duration, and number of saccades—all of which decreased significantly under time-constrained conditions. This suggests that athletes tend to adopt more simplified and direct visual search strategies under high-pressure scenarios, aiming to conserve cognitive resources and rapidly identify critical targets. These findings are consistent with those of Lindong et al. (2016), who observed that time pressure leads to a reduction in search duration in eye-tracking experiments. Moreover, the decrease in fixation frequency and duration indicates that, when time is limited, individuals may sacrifice depth of information processing, increasing the risk of cognitive bias and judgment errors—an observation that may partly explain the drop in decision-making accuracy under time pressure. Interestingly, while gaze heatmaps and scanpath diagrams became more scattered under pressure, this effect was more pronounced in the novice group. Expert players maintained relatively focused visual attention patterns, whereas novices exhibited significantly more dispersed search behaviors. This discrepancy can be attributed to the cognitive advantages developed through long-term systematic training and competitive experience. Expert athletes have likely formed stable sport-specific long-term memory structures, which enable them to rapidly identify key cues and efficiently process information with minimal visual input. Furthermore, their frequent exposure to high-pressure competitive environments has enhanced their psychological regulation capabilities. According to the attentional narrowing theory under time pressure, time constraints drive individuals to focus attention on task-relevant information—a process known as “task-focused attention”—which helps optimize performance outcomes (Kelly and Loving, 2004). Experts, with their robust long-term memory and cognitive schemas, are better equipped to allocate attentional resources effectively and filter out irrelevant stimuli, thereby sustaining high-level performance under pressure. In contrast, novice players lack such stable memory support and sport-specific experience. When faced with time-pressured tasks, they are unable to compensate for limited information-processing capacity by increasing fixations or saccades. This results in imbalanced attentional allocation, missed critical cues, heightened cognitive load, and ultimately a decline in task performance. The findings of this study are in line with previous research by Linchao (2020), who reported that high-level orienteering athletes exhibited more stable eye movement patterns and greater behavioral efficiency under time pressure, whereas lower-level athletes were more adversely affected.

### 4.3 Effect size and practical application

Although the interaction between time pressure and skill level reached statistical significance in terms of saccade count, fixation duration, and fixation count, indicating significant differences in the visual behaviors of athletes with different skill levels under time pressure, the effect size was small. This suggests that while the difference is statistically reliable, its impact in real-game situations may be limited. Therefore, coaches and athletes should consider this difference as a relatively minor factor in practical applications. Although more efficient visual search may contribute to improved decision-making efficiency (Piras, 2025), the effect of visual search alone might not lead to significant changes in actual gameplay. Only through the integration of more scientifically grounded visual search strategies can better outcomes be achieved. From the gaze trajectory and heatmap analysis, it is evident that the expert group exhibited more focused and logical gaze patterns, with more relevant observation areas for decision-making, leading to higher decision quality. This finding provides valuable insights for coaches, athletes, and referees, helping them infer and optimize cognitive processes such as attention allocation, decision-making, and prediction. In turn, this can inform the development of targeted tactical plans and attention distribution strategies for key areas, thereby improving athletic performance. It is worth noting that the small effect size may also be due to other factors. For example, time pressure may consume significant cognitive resources and negatively affect athletes’ psychological states, leading to anxiety and discomfort (Yan-qing et al., 2022). These psychological factors may also influence decision-making. Previous research has demonstrated that psychological states, emotions, and other external factors play a crucial role in the decision quality of athletes (Lulu et al., 2024; Yaoyao, 2024).

### 4.4 Limitations and future directions

This study highlights the potential of eye-tracking technology in the field of sports science, offering feasible methods for future research. By gaining deeper insights into athletes’ visual behaviors, coaches and researchers can better guide training and optimize performance strategies. However, several limitations should be acknowledged: (1) Ecological Validity of the Experimental Task Needs Improvement: This study employed video simulation tasks to present competitive scenarios. While this approach helps control variables, it still fails to fully capture the dynamic interactions and physical execution inherent in actual gameplay. To enhance ecological validity, future research should prioritize the use of mobile eye-tracking devices to study visual behaviors in more ecologically valid settings. This will provide a more comprehensive understanding of how athletes, coaches, and referees perform under the pressure of live competition, drawing on AI systems developed by Lozzi et al. (2025). Additionally, combining eye-tracking technology with other physiological and cognitive measures would enable a more thorough exploration of the factors influencing basketball performance. (2) Limitations of the Eye-Tracking Equipment Used: The eye tracker used in this study was non-wearable, which limits its ability to measure changes in pupil size, thus restricting the measurement of cognitive load. To more accurately assess the impact of time pressure on decision-making, future studies should consider using wearable eye-tracking devices, in combination with EEG, pupillometry, and heart rate variability (HRV) to quantitatively measure athletes’ cognitive load from multiple dimensions. This approach would provide a more thorough investigation of the relationship between pressure and decision-making. (3) Small Effect Size Despite Significant Interaction Effects: Although significant interaction effects were found in this study, the small effect size suggests that the practical impact of the inter-group differences may be limited. Future studies could incorporate individual difference analysis to explore how different psychological states, cognitive load, and physiological responses (such as heart rate and skin conductance) influence athletes’ decision-making under time pressure. Through these deeper analyses, more subtle individual differences can be revealed, which will further enhance the practical relevance and value of the research.

## 5 Conclusion

(1) Athletes at different skill levels exhibit significant differences in visual search behavior. Expert players demonstrated more efficient visual search strategies, faster reaction times, and superior decision-making performance. (2) Under time pressure, expert athletes maintained relatively stable decision accuracy despite faster responses. Their eye movement patterns remained consistent, indicating strong adaptive capabilities in high-pressure situations. (3) In contrast, novice players showed a marked decline in decision accuracy and reduced visual search efficiency under time constraints, reflecting weaker cognitive regulation abilities when faced with performance pressure.

## Data Availability

The original contributions presented in the study are included in the article/Supplementary material, further inquiries can be directed to the corresponding author.

## References

[ref1] Alemanno M. Di Pompeo I. Marcaccio M. Canini D. Curcio G. Migliore S. (2025). From gaze to game: a systematic review of eye-tracking applications in basketball. Brain Sci. 15:421. doi: 10.3390/brainsci15040421, PMID: 40309899 PMC12025553

[ref2] Bourgeais Q. Charrier R. Sanlaville E. Seifert L. (2025). Temporal passing network in basketball: the effect of time pressure on the dynamics of team organization at micro and meso levels. Psychol. Sport Exerc. 80:102882. doi: 10.1016/j.psychsport.2025.102882 40403945

[ref3] Dawei W. (2009). The effect of time stress on decision making. Psychol. Res. 2, 42–46. doi: 10.7666/d.y1073781

[ref4] Dawei W. (2010). The effect of time pressure and task type on the decision-making strategy. Chin. J. Spec. Educ., 79–83. doi: 10.3969/j.issn.1007-3728.2010.10.016

[ref5] Du L. Chen Y. (2023). Does time stressor promote or inhibit employees’ proactive work behavior? Cognitive appraisals of stress as mediator and time management skills as moderator. Hum. Resour. Dev. China 40, 6–20. doi: 10.16471/j.cnki.11-2822/c.2023.4.001

[ref6] Giancamilli F. Galli F. Chirico A. Fegatelli D. Mallia L. Palombi T.. (2022). When the going gets tough, what happens to quiet eye? The role of time pressure and performance pressure during basketball free throws. Psychol. Sport Exerc. 58:102057. doi: 10.1016/j.psychsport.2021.102057

[ref7] Hsiao S.-W. Wang M.-F. Chen C.-W. (2017). Time pressure and creativity in industrial design. Int. J. Technol. Des. Educ. 27, 271–289. doi: 10.1007/s10798-015-9343-y

[ref8] Hu H. (2017). Common types and countermeasures of ankle ligament injury caused by intense basketball movement. Niger. J. Clin. Pract. 20, 1036–1039. doi: 10.4103/njcp.njcp_145_16, PMID: 28891550

[ref9] Jiang Y. Huang P. Qian X. (2024). Reducing the influence of time pressure on risky choice the role of cognitive reappraisal. Exp. Psychol. 71, 238–246. doi: 10.1027/1618-3169/a000626, PMID: 39552412

[ref11] JiaRan J. (2024). Visualization of basketball tactical training system based on eye tracking technology [master’s degree in Chinese]. Available online at: https://link.cnki.net/doi/10.27267/d.cnki.gqfsu.2024.001301

[ref10] Jin P. Ge Z. Fan T. (2023). Research on visual search behaviors of basketball players at different levels of sports expertise. Sci. Rep. 13:1406. doi: 10.1038/s41598-023-28754-2, PMID: 36697486 PMC9876905

[ref12] Kelly J. R. Loving T. J. (2004). Time pressure and group performance: exploring underlying processes in the attentional focus model. J. Exp. Soc. Psychol. 40, 185–198. doi: 10.1016/s0022-1031(03)00094-5

[ref13] Kocher M. G. Schindler D. Trautmann S. T. Xu Y. (2019). Risk, time pressure, and selection effects. Exp. Econ. 22, 216–246. doi: 10.1007/s10683-018-9576-1

[ref14] Li Y. Feng T. Zhang F. Asgher U. Yan B. Peng T. (2023). Visual search strategies of performance monitoring used in action anticipation of basketball players. Brain Behav. 13:e3298. doi: 10.1002/brb3.3298, PMID: 37872861 PMC10726756

[ref15] Li A. Yan L. Wang X. Ma X. Li F. (2015). The double-edged effect and mechanism of time pressure. Adv. Psychol. Sci. 23, 1627–1636. doi: 10.3724/SP.J.1042.2015.01627

[ref16] Linchao Y. (2020). Research on visual search characteristics of directional map recognition under time pressure [master’s degree in Chinese]. Available online at: https://link.cnki.net/doi/10.27280/d.cnki.gsdsu.2020.000570

[ref17] Lindong Y. Fuxuan S. Yunhong Z. Ruifeng Y. (2016). Effects of target prevalence, target presence and time pressure on visual search performance. Ind. Eng. J. 19, 83–122. doi: 10.3969/j.issn.1007-7375.2016.06.013

[ref18] Liu M. Kong A. Lau N. Feng Z. Liu X. (2024). Basketball self-evaluation matrix: discrepancy between self-confidence and decision-making performance on psychological profiling of players. Front. Sports Act. Living 6:1404701. doi: 10.3389/fspor.2024.1404701 39444493 PMC11496061

[ref19] Lozzi D. Di Pompeo I. Marcaccio M. Alemanno M. Krüger M. Curcio G.. (2025). AI-powered analysis of eye tracker data in basketball game. Sensors 25:3572. doi: 10.3390/s25113572 40719537 PMC12158319

[ref20] Lulu S. Shiguang W. Jiahui C. Ping R. (2024). A study on the influence of emotional state and risk-taking tendency on offensive decision-making in basketball players. J. Beijing Sport Univ. 47, 124–134. doi: 10.19582/j.cnki.11-3785/g8.2024.11.011

[ref21] Nakamoto H. Mori S. (2008). Sport-specific decision-maklng in a Go/NoGo reaction task: difference among nonathletes and baseball and basketball players. Percept. Mot. Skills 106, 163–170. doi: 10.2466/pms.106.1.163-170 18459365

[ref22] Nian Q. Lu W. Xu Y. (2023). Effects of object working memory load on visual search in basketball players: an eye movement study. BMC Psychol. 11:446. doi: 10.1186/s40359-023-01488-6, PMID: 38115097 PMC10731696

[ref23] Pearsall M. J. Ellis A. P. J. Stein J. H. (2009). Coping with challenge and hindrance stressors in teams: behavioral, cognitive, and affective outcomes. Organ. Behav. Hum. Decis. Process. 109, 18–28. doi: 10.1016/j.obhdp.2009.02.002

[ref24] Peng S. Guodong W. Zheng W. Jinyue S. Dongyang Z. Duoyao Z. (2023). Visual search characteristics of football players in the context of offensive tactical pre-judgment decision-making: effect of spatial working memory capacity. China Sport Sci. Technol. 59, 27–34. doi: 10.16470/j.csst.2022079

[ref25] Piras A. (2025). The role of the peripheral target in stimulating eye movements. Psychol. Sport Exerc. 76:102744. doi: 10.1016/j.psychsport.2024.102744 39307329

[ref26] Quan F. (2004). Summary of researches on decision-making in sports. J. Beijing Sport Univ., 863–865. doi: 10.19582/j.cnki.11-3785/g8.2004.06.053

[ref27] Sha H. (2022). Experimental study of visual search characteristics of college basketball players under time pressure [master’s degree in Chinese]. Available online at: https://link.cnki.net/doi/10.27410/d.cnki.gxbfu.2022.001814

[ref28] Sireteanu R. Rettenbach R. (1995). Perceptual learning in visual search: fast, enduring, but non-specific. Vis. Res. 35, 2037–2043. doi: 10.1016/0042-6989(94)00295-w, PMID: 7660607

[ref29] Wenfang S. Xinyue W. Changsheng W. Ming Z. Bin W. (2018). Visual search feature of expert athletes: a meta-analysis of eye-tracking studies. J. Tianjin Univ. Sport 33, 321–328. doi: 10.13297/j.cnki.issn1005-0000.2018.04.007

[ref31] Xian-yu W. Hong-ting L. Shu M. (2021). Research progress on factors affecting icon search performance. Packaging Eng. 42, 206–211. doi: 10.19554/j.cnki.1001-3563.2021.06.029

[ref30] XiaoFei M. (2024). The effect of time stress on the effect of risk decision-making frameworks: based on evidence from eye tracking experiments [master’s degree in Chinese]. Available online at: https://link.cnki.net/doi/10.27262/d.cnki.gqdau.2024.002852

[ref32] Xiaoting W. Lizhong C. Pengfei R. (2022). Verbal working memory capacity moderates the influence of mind wandering on decision-making in badminton. J. Tianjin Univ. Sport 37, 489–496. doi: 10.13297/j.cnki.issn1005-0000.2022.04.017

[ref33] Xiuying M. Lizhong C. (2017). The research of unconscious thought in sport decision making. J. Psychol. Sci. 40, 329–334. doi: 10.16719/j.cnki.1671-6981.20170212

[ref34] XuYang L. (2024). ERP study on sports decision-making of football players with different spatial working memory capacities under time pressure [master’s degree in Chinese]. Available online at: https://link.cnki.net/doi/10.27384/d.cnki.gwhtc.2024.000141

[ref35] Yan-qing W. Shi-qi Z. Rui L. (2022). Emotional stability of student pilots under different time pressure conditions decision-making performance. Sci. Technol. Eng., 22, 5513–5518. doi: 10.3969/j.issn.1671-1815.2022.13.053

[ref36] Yaoyao W. (2024). Role analysis and operating mechanism of emotions in decision-making paradigms. J. Sichuan Normal Univ. 51, 52–58 +200. doi: 10.13734/j.cnki.1000-5315.2024.0205

[ref37] Yirui S. Changzhu Q. Congyi W. Jialiang L. (2024). Advantageous characteristics and neural mechanisms of temporal duration perception in table tennis athletes evidenced by ERP and EEG. China Sport Sci. Technol. 60, 23–30. doi: 10.16470/j.csst.2024095

[ref38] Yuan C. Yang Y. Liu Y. (2021). Sports decision-making model based on data mining and neural network. Neural Comput. & Applic. 33, 3911–3924. doi: 10.1007/s00521-020-05445-x

[ref39] Yunchao M. Mengyao R. Xingman L. (2023). Application of virtual simulation technology in sports decision training: a systematic review. Front. Psychol. 14:1164117. doi: 10.3389/fpsyg.2023.1164117 37275736 PMC10232800

[ref40] Zheng L. Mengshi X. Lei H. (2019). The influence of materialism and time pressure on risky decision-making. J. Psychol. Sci. 42, 1422–1427. doi: 10.16719/j.cnki.1671-6981.20190621

