# Supplementary material for: The impact of time pressure on decision-making and visual search characteristics in basketball players
Source: Front Psychol. 2025 Aug 13;16:1660732. doi: 10.3389/fpsyg.2025.1660732 (PMC12380712; doi:10.3389/fpsyg.2025.1660732)
Supplement: Supplementary file 1 [file Supplementary_file_1.doc]

Table S1 Information of Study Participants

| Group | Number of Participants | Athletic Level | Training Experience (Years) | Age |
| --- | --- | --- | --- | --- |
| Expert group | 20 | National First-Class or Higher-Level Athletes | 9.10±0.72 | 20.15±1.18 |
| Novice group | 20 | National second-level | 4.05±0.83 | 19.50±1.43 |

Table S2 Time Pressure Scale

| Question | Completely inconsistent | Inconsistent | | Basically consistent | Quite consistent | Completely consistent |
| --- | --- | --- | --- | --- | --- | --- |
| 1. I feel very nervous or anxious | 1 | 2 | 3 | | 4 | 5 |
| 2. I feel that time passes quickly | 1 | 2 | 3 | | 4 | 5 |
| 3.  I feel that there is not enough time | 1 | 2 | 3 | | 4 | 5 |
| 4.  I feel that my heart is beating faster | 1 | 2 | 3 | | 4 | 5 |
| 5. I keep checking the time | 1 | 2 | 3 | | 4 | 5 |
| 6. I feel quite upset | 1 | 2 | 3 | | 4 | 5 |
| 7. I think I simply can't finish the task within the limited time | 1 | 2 | 3 | | 4 | 5 |
| 8. Time limits my attention | 1 | 2 | 3 | | 4 | 5 |

Table S3 Descriptive Statistics of Time Pressure Test

| Group | No time pressure | With time pressure |
| --- | --- | --- |
| Experts | 1.42±0.15 | 3.08±0.18 |
| Novices | 1.70±0.21 | 3.40±0.16 |

Table S4 Repeated-measures ANOVA results for time pressure across basketball players with different skill levels

| Source of variance | df | F | P | η²ₚ |
| --- | --- | --- | --- | --- |
| Time pressure | 1 | 2464.916 | 0.001** | 0.985 |
| Skill level | 1 | 45.508 | 0.001** | 0.545 |
| Skill Level × Time Pressure | 1 | 0.131 | 0.720 | 0.003 |

**Table S5 Descriptive Statistics of Accuracy Results of Expert - Novice Basketball athletes(M±SD)**

| Group / With or without time pressure | No time pressure | With time pressure |
| --- | --- | --- |
| Expert group | 0.786±0.023 | 0.734±0.022 |
| Novice group | 0.697±0.054S | 0.602±0.066 |

Table S6 Descriptive Statistics of Reaction Time of Expert - Novice Basketball athletes (M±SD)

| Group / With or without time pressure | No time pressure（ms） | With time pressure（ms） |
| --- | --- | --- |
| Expert group | 2169.10±116.15 | 1302.15±89.72 |
| Novice group | 2078.95±205.09 | 1849.30±122.95 |

Table S7 Descriptive Statistics of Fixation Counts of Expert - Novice Basketball athletes (M±SD)

| Group / With or without time pressure | No time pressure（count） | With time pressure（count） |
| --- | --- | --- |
| Expert group | 7.05±0.94 | 5.15±0.59 |
| Novice group | 10.30±1.34 | 7.35±0.67 |

### Table S8 Descriptive Statistics of Fixation Duration of Expert - Novice Basketball athletes (M±SD)

| Group / With or without time pressure | No time pressure（ms） | With time pressure（ms） |
| --- | --- | --- |
| Expert group | 2132.30±225.78 | 1461.80±221.57 |
| Novice group | 2541.65±138.80 | 1644.65±300.10 |

Table S9 Descriptive Statistics of Saccade Counts of College Basketball athletes at Different Levels (M±SD)

| Group / With or without time pressure | No time pressure（count） | With time pressure（count） |
| --- | --- | --- |
| Expert group (21) | 6.95±0.94 | 4.40±0.88 |
| Novice group (21) | 9.15±0.99 | 5.85±0.93 |
